# Hepatic NAPE-PLD Is a Key Regulator of Liver Lipid Metabolism

**DOI:** 10.3390/cells9051247

**Published:** 2020-05-18

**Authors:** Charlotte Lefort, Martin Roumain, Matthias Van Hul, Marialetizia Rastelli, Rita Manco, Isabelle Leclercq, Nathalie M. Delzenne, Vincenzo Di Marzo, Nicolas Flamand, Serge Luquet, Cristoforo Silvestri, Giulio G. Muccioli, Patrice D. Cani

**Affiliations:** 1 Metabolism and Nutrition Research Group, Louvain Drug Research Institute, Walloon Excellence in Life sciences and BIOtechnology (WELBIO), UCLouvain, Université Catholique de Louvain, Av. E. Mounier, 73 B1.73.11, 1200 Bruxelles, Belgium; charlotte.lefort@uclouvain.be (C.L.); matthias.vanhul@uclouvain.be (M.V.H.); marialetizia.rastelli@uclouvain.be (M.R.); nathalie.delzenne@uclouvain.be (N.M.D.); 2 Bioanalysis and Pharmacology of Bioactive Lipids Research Group, Louvain Drug Research Institute, UCLouvain, Université Catholique de Louvain, 1200 Bruxelles, Belgium; martin.roumain@uclouvain.be (M.R.); giulio.muccioli@uclouvain.be (G.G.M.); 3 Laboratory of Hepato-Gastroenterology, UCLouvain, Université catholique de Louvain, 1200 Bruxelles, Belgium; rita.manco@weizmann.ac.il (R.M.); isabelle.leclercq@uclouvain.be (I.L.); 4 Quebec Heart and Lung Institute Research Centre, Université Laval, Quebec City, QC G1V 0A6, Canada; vincenzo.dimarzo@criucpq.ulaval.ca (V.D.M.); nicolas.flamand@criucpq.ulaval.ca (N.F.); cristoforo.silvestri@criucpq.ulaval.ca (C.S.); 5 Centre NUTRISS, Institute of Nutrition and Functional Foods, Université Laval, Quebec City, QC G1V 0A6, Canada; 6 Endocannabinoid Research Group, Institute of Biomolecular Chemistry, Consiglio Nazionale delle Ricerche, 80078 Pozzuoli, Napoli, Italy; 7 Université de Paris, BFA, UMR 8251, CNRS, F-75014 Paris, France; serge.luquet@univ-paris-diderot.fr

**Keywords:** NAPE-PLD, NAEs, bioactive lipids, bile acids, inflammation, liver, obesity

## Abstract

Diverse metabolic disorders have been associated with an alteration of *N*-acylethanolamine (NAE) levels. These bioactive lipids are synthesized mainly by *N*-acylphosphatidylethanolamine-selective phospholipase D (NAPE-PLD) and influence host metabolism. We have previously discovered that NAPE-PLD in the intestine and adipose tissue is connected to the pathophysiology of obesity. However, the physiological function of NAPE-PLD in the liver remains to be deciphered. To study the role of liver NAPE-PLD on metabolism, we generated a new mouse model of inducible *Napepld* hepatocyte-specific deletion (*Napepld*^∆Hep^ mice). In this study, we report that *Napepld*^∆Hep^ mice develop a high-fat diet-like phenotype, characterized by an increased fat mass gain, hepatic steatosis and we show that *Napepld*^∆Hep^ mice are more sensitive to liver inflammation. We also demonstrate that the role of liver NAPE-PLD goes beyond the mere synthesis of NAEs, since the deletion of NAPE-PLD is associated with a marked modification of various bioactive lipids involved in host homeostasis such as oxysterols and bile acids. Collectively these data suggest that NAPE-PLD in hepatocytes is a key regulator of liver bioactive lipid synthesis and a dysregulation of this enzyme leads to metabolic complications. Therefore, deepening our understanding of the regulation of NAPE-PLD could be crucial to tackle obesity and related comorbidities.

## 1. Introduction

Over the last few years, interest in specific bioactive lipids, the *N*-acylethanolamines (NAEs), has increased exponentially as accumulating evidence demonstrated an association between variations in NAE levels and diverse pathological conditions such as obesity, inflammation or hepatic disorders [1,2,3]. NAEs belong to the expanded endocannabinoid system, or endocannabinoidome (eCBome), which is composed of several enzymes, receptors and bioactive lipids involved in energy homeostasis [2,4]. The best-known NAEs include anandamide (*N*-arachidonoylethanolamine, AEA, an endocannabinoid), *N*-palmitoylethanolamine (PEA), *N*-stearoylethanolamine (SEA), *N*-linoleylethanolamine (LEA) and *N*-oleoylethanolamine (OEA). In the context of metabolic disorders, some NAEs have been reported to regulate food intake [5,6,7], to mediate inflammation [8,9] or to contribute to the development of steatosis [10,11]. Even though many of their physiological actions have been discovered, understanding the molecular mechanisms that link NAEs to metabolic diseases remains challenging.

Since *N*-acylphosphatidylethanolamine-selective phospholipase D (NAPE-PLD) is the main enzyme producing NAEs [12], inhibiting its action, represents an efficient way to explore the function of NAEs. However, the mechanisms of regulation of NAPE-PLD activity, especially in vivo, are not yet fully deciphered. Interestingly, recent studies have made progress in that perspective and have elegantly shown that natural bile acids, and specific steroidal hydroxylation pattern were key elements for modulating the enzyme activity [13]. These data suggesting a link between NAPE-PLD and steroid acids open the floor to design putative small-molecule modulators with potential therapeutic applications [14].

Nevertheless, in order to better understand the physiological role of NAPE-PLD, deleting this enzyme in the whole-body has been the initial strategy. Although whole-body *Napepld*-knockouts displayed controversial brain lipid profiles, they did not exhibit a peculiar phenotype [15,16,17,18,19], suggesting that compensatory mechanisms come into effect when this vital system is deficient in early developmental stages. To overcome this problem, we generated *Napepld*-deleted mouse models in which NAPE-PLD could be inactivated in specific organs from adult mice, using the Cre-lox system [20]. This allowed us to previously discover that mice deleted for *Napepld* in the adipose tissue developed an obese-like phenotype, with higher fat mass, glucose intolerance and low-grade inflammation, when fed a normal diet [21]. In addition, we also demonstrated that the deletion of *Napepld* in the intestinal epithelial cells affected food intake regulation [22]. These two studies highlighted that NAPE-PLD and the bioactive products it generates are involved in host homeostasis and that the phenotypes observed were organ-specific. Interestingly, in humans, a single nucleotide polymorphism in *Napepld* has been linked to obesity [23]. Since obesity is associated with a cluster of metabolic complications including liver disorders [24,25,26], and both organ-specific *Napepld* knockout mouse models previously investigated were prone to develop obesity-associated steatosis, we next wondered what would be the role of NAPE-PLD in the liver. In the present study, we therefore generated a new mouse model of inducible hepatocyte-specific deletion of *Napepld* in hepatocytes (*Napepld*^∆Hep^) to further investigate the physiological functions of this enzyme in the context of metabolic diseases.

## 2. Materials and Methods

### 2.1. Mice

All mice were bred in our specific opportunistic and pathogen free (SOPF) animal facility. Animals were housed filter-top cages kept in a controlled environment (room temperature of 22 ± 2 °C, humidity 55% ± 10%, 12 h daylight cycle) in groups of two mice per cage, with free access to irradiated food and autoclaved water. Mice were fed a normal diet (ND; AIN93Mi, Research Diets, New Brunswick, NJ, USA). Body composition (lean and fat mass) was assessed by using 7.5 MHz time domain-nuclear magnetic resonance (TD-NMR; LF50 Minispec, Bruker, Rheinstetten, Germany). This technique allows one to follow in vivo the development of adipose tissue, fat-free mass in non-anesthetized mice throughout the experimental treatment.

### 2.2. Generation of Napepld^∆Hep^ Mice

Inducible hepatocyte *Napepld*-deleted C57Bl6/J mice (*Napepld*^∆Hep^) were generated by crossing mice harboring a tamoxifen-dependent Cre recombinase expressed under the control of the albumin promoter (Albumin Cre-ERT2 mice were kindly provided by Prof. Pierre Chambon, GIE-CERBM (IGBMC), Illkirch, France) with mice bearing a *loxP*-flanked *Napepld* allele [21,27]. After verification of the genotype by PCR, littermate mice were randomly assigned to the different experimental groups. The deletion was induced at 8 weeks of age by intra-peritoneal (i.p.) injection of 100 μL tamoxifen (10 mg/mL) for 5 consecutive days. The control mice (wild-type (WT) that is *Napepld*^lox/lox^ × Albumin Cre-ERT2) were injected by an i.p. injection of 100 μL of vehicle (filtered sunflower oil with ethanol) for 5 consecutive days. Tamoxifen was prepared by addition of ethanol to 100 mg of tamoxifen (tamoxifen-free base, MP Biomedicals) to obtain a 100 mg/mL of tamoxifen suspension. A 10 mg/mL tamoxifen solution was prepared by addition of filtered sunflower oil, followed by 30 min sonication. The 10 mg/mL solution of tamoxifen solution was stored at 4 °C for up to 1 week and was sonicated 5 min before use. After the 5 days of injections, mice were allowed to rest for two weeks to recover from any stress or discomfort they may have experienced due to the injections and to allow any residual oil and ethanol to wash out.

### 2.3. Phenotyping in Normal Diet (ND) Condition

A cohort of 11-week-old male *Napepld*^∆Hep^ and WT mice were fed a control diet (AIN93Mi, Research Diets) for 7 weeks.

### 2.4. Phenotyping in High-Fat Diet (HFD) Condition

A cohort of 11-week-old male *Napepld*^∆Hep^ and WT mice were fed a HFD (60% fat, D12492i, Research Diets) for 8 weeks.

### 2.5. Oral Glucose Tolerance Test

One week before the end of the experiments, mice were fasted for 6 h prior to be given an oral glucose load (2 g glucose per kg body weight). Blood glucose levels were measured 30 min before, just prior to gavage, and 15, 30, 60, 90 and 120 min after oral glucose load using a standard glucose meter (Accu Check, Roche, Basel, Switzerland) on the tip of the tail vein. Plasma samples were collected from the tip of the tail vein in heparinized tubes 30 min before and 15 min after oral glucose load for determination of insulin concentration.

### 2.6. Insulin Resistance Index

Plasma insulin concentration was determined using an ultrasensitive mouse insulin ELISA kit (Mercodia, Uppsala, Sweden) according to the manufacturer’s instructions. Insulin resistance index was determined by multiplying the area under the curve of both blood glucose (−30 and 15 min) and plasma insulin (−30 and 15 min) obtained following the oral glucose tolerance test.

### 2.7. LPS Injection Experiment

A cohort of 14-week-old ND-fed male *Napepld*^∆Hep^ and WT mice were injected, as previously described [28], intraperitoneally with either 300 µg/kg LPS solution (LPS from *Escherichia coli* O55:B5; Sigma L2880) or saline solution (CT). Mice were killed 4 h after the injection.

### 2.8. Tissue Sampling

At the end of the experiment, mice were anesthetized with isoflurane (Forene, Abbott, Queenborough, Kent, England) after a fasting period of 6 h. Blood was sampled from the portal and cava veins. After exsanguination, mice were killed by cervical dislocation. Tissues were precisely dissected, weighed and immediately immersed in liquid nitrogen followed by storage at −80 °C for further analysis.

### 2.9. Hepatocyte Isolation

A cohort of 11-week-old ND-fed male *Napepld*^∆Hep^ and WT mice were anesthetized with Ketamine-Xylazine (Nimatek, Eurovet Animal Health BV, Bladel, Netherlands-Rompun, Bayer Healthcare, Loos, France) solution. The mice were surgically opened and the liver was infused with a PBS solution containing EGTA and HEPES buffer injected in the portal vein via a 26 g needle at a rate of 9 mL/min for 10 min. Warm (37 °C) digestion medium including DMEM/F12, no glutamine (Thermo Fisher, Waltham, MA, USA), Penicillin-Streptomycin, HEPES buffer, thermolysin (Promega, Madison, WI, USA), collagenase G (Abiel, Palermo, Italy) and collagenase H (Abiel, Palermo, Italy) was then added for 7 min. The liver was then removed and incubated with the medium at 37 °C for 15 min for further digestion. The obtained solution was then filtered (70 µm) and centrifuged twice (4 °C, 50 g, 2 min) to allow the separation between hepatocytes and non-parenchymal cells (NPC). The resulting pellet contained the hepatocytes whereas NPC were enriched in the supernatant. After isolating the two fractions in different tubes, they were centrifuged (4 °C, 10,000× *g*, 10 min) and pellets were stored in −80 °C prior TriPure RNA extraction. 

### 2.10. RNA Preparation and Real-Time qPCR Analysis

Total RNA was prepared from tissues using TriPure reagent (Roche Diagnostics, Penzberg, Germany). Quantification and integrity analysis of total RNA were performed by analyzing 1 μL of each sample in an Agilent 2100 Bioanalyzer (Agilent RNA 6000 Nano Kit, Agilent). cDNA was prepared by reverse transcription of 1 μg total RNA using the GoScript Reverse Transcriptase kit (Promega, Madison, WI, USA). Real-time PCR was performed with the QuantStudio 3 real-time PCR system (Thermo Fisher, Waltham, MA, USA). *Rpl19* RNA was chosen as the housekeeping gene. All samples were performed in duplicate, and data were analyzed according to the 2^−ΔΔCT^ method. The identity and purity of the amplified product were assessed by melting curve analysis at the end of amplification. The primer sequences for the targeted mouse genes are presented in Table 1.

### 2.11. Adipocyte Histological Analysis

SAT tissues were fixed in 4% formaldehyde. Hematoxylin and eosin staining was performed using standard protocols on 5-µm adipose tissue sections. Adipocyte size was determined using ImageJ software (version 1.50a, National Institutes of Health, Bethesda, MD, USA).

### 2.12. Hepatic Lipid Content Analysis by Oil Red O Staining

Liver tissue was embedded in Tissue-Tek Optimal Cutting Temperature compound (Sakura Europe, Leiden, Netherlands) and flash-frozen in cold isopentane. Five µm-tick tissue sections were stained with oil red O staining for lipid content analysis. Quantification of the mean droplet size was performed using ImageJ software (version 1.50a, National Institutes of Health, Bethesda, MD, USA).

### 2.13. Extraction of Liver Lipids

Total lipids were measured in the liver tissue after extraction in CHCl_3_:MeOH according to the Folch method [29] and adapted as follows: briefly, 100 mg of liver tissue was homogenized in 2 mL of CHCl_3_:MeOH (2:1) using a tissue lyser followed by an ultrasonic homogenizer. Four hundred microliter of 0.9% NaCl solution was added and lipids were then extracted by vigorous shaking. After centrifugation, the chloroform phase was recovered in glass tubes and dried under a stream of N2. Glass tubes were weighed before and after lipid extraction to quantify total lipid content. The dried residue was solubilized in 1.5 mL isopropanol.

### 2.14. Biochemical Analyses

Plasma non-esterified fatty acids (NEFA) and liver and plasma cholesterol and triglyceride concentrations were measured using kits coupling an enzymatic reaction with spectrophotometric detection of the reaction endproducts (Diasys Diagnostic and Systems, Holzheim, Germany) according to the manufacturer’s instructions.

### 2.15. Lipidomics Analysis

All lipids measured are presented in Table 2.

*eCBome mediators*. NAEs and other eCBome mediators were quantified by LC–MS/MS as described in Everard, Plovier and Rastelli et al. [22]. Briefly, liver lipids were injected in the HPLC system interfaced with the electrospray source of a Shimadzu 8050 triple quadrupole mass spectrometer and mass spectrometric analysis was done in the positive ion mode using multiple reaction monitoring using the specific mass transitions [30].

*Oxysterols*. Oxysterols were analyzed by LC-MS as previously reported [31]. Briefly, lipids were extracted, in the presence of internal standards, through liquid–liquid extraction, and then samples were prepurified by solid phase extraction. The eluate was recovered and injected in the LC–MS system consisting in an Accela HPLC system (Thermo Fisher Scientific) coupled to an LTQ-Orbitrap XL mass spectrometer (Thermo Fisher Scientific) equipped with an APCI probe used in positive mode. Lipids were quantified using calibration curves obtained using the same procedure.

*Bile acids*. Bile acids were quantified using a LC-MS method adapted from Guillemot-Legris et al. [32]. Liver samples were homogenized in ice-cold bi-distilled water. Proteins were then precipitated overnight at −20 °C using acetone containing deuterated internal standards. Supernatant was recovered and evaporated to dryness. The organic residue was resuspended in methanol and injected in the LC–MS system, equipped with an electrospray probe used in negative mode. Lipids were quantified using calibration curves obtained using the same procedure.

### 2.16. Ethics Statement

All mouse experiments were reviewed and approved by and performed in accordance with the guidelines of the local ethics committee for animal care of the Health Sector of the Université catholique de Louvain under the specific agreement numbers 2014/UCL/MD/022 and 2017/UCL/MD/005. Housing conditions were as specified by the Belgian Law of 29 May 2013 regarding the protection of laboratory animals (agreement number LA1230314). Every effort was made to minimize animal pain, suffering, and distress.

### 2.17. Statistical Analysis

Data were presented as the mean ± s.e.m. The difference between two groups was evaluated by a *t*-test. Differences between more than two groups were assessed by two-way ANOVA, followed by the Tukey post hoc test. Data were analyzed with GraphPad Prism (GraphPad Software).

## 3. Results

### 3.1. Hepatocyte-Specific Deletion of Napepld

**Figure 1:**
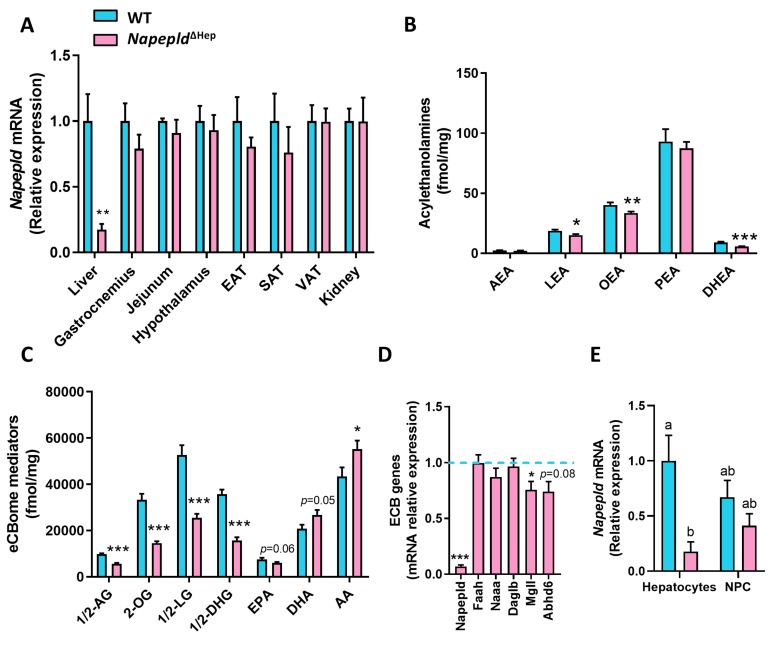
Hepatocyte-specific deletion of *Napepld*. (**A**) *Napepld* mRNA expression measured by real-time qPCR in the liver, gastrocnemius muscle, jejunum, hypothalamus, epididymal adipose tissue (EAT), subcutaneous adipose tissue (SAT), visceral adipose tissue (VAT) and kidney (*n* = 6). (**B–C**) Levels of *N*-acylethanolamines and other eCBome mediators in the liver (fmol/mg; *n* = 8–10). (**D**) mRNA expression of key enzymes of the endocannabinoid system (ECB) measured by real-time qPCR in the liver (*n* = 8–10). (**E**) *Napepld* mRNA expression in hepatocytes and hepatic non-parenchymal cells (NPC; *n* = 4–5). Blue: wild-type (WT) normal diet (ND) mice. Pink: *Napepld*^∆Hep^ ND mice. Data are presented as the mean ± s.e.m. *, ** and *** indicate a significant difference versus WT ND (Respectively *p* < 0.05, *p* < 0.01 and *p* < 0.001) according to the *t*-test. Data with different superscript letters are significantly different (*p* < 0.05) according to two-way ANOVA followed by Tukey post hoc test. Lipid abbreviations: 1/2-AG, 1/2-arachidonoylglycerol; 1/2-DHG, 1/2-DHA-glycerol; 1/2-LG, 1/2-linoleoylglycerol; 2-OG, 2-oleoylglycerol; AA, Arachidonic acid; AEA, *N*-arachidonoylethanolamine; DHA, Docosahexaenoic acid; DHEA, *N*-docosahexaenoylethanolamine; EPA, Eicosapentaenoic acid; LEA, *N*-linoleylethanolamine; OEA, *N*-oleoylethanolamine; PEA, *N*-palmitoylethanolamine.

To explore the role of liver NAPE-PLD on metabolism, *Napepld*^∆Hep^ mice were generated by crossing Albumin-Cre^ERT2^ mice [27] with *Napepld*^lox/lox^ mice [21]. The liver specificity of the deletion upon tamoxifen treatment was assessed by quantifying *Napepld* messenger RNA (mRNA) expression in the liver and in several organs including adipose tissues, brain, intestine, muscle or the kidney of wild-type (WT) and *Napepld*^∆Hep^ mice fed a control diet (Figure 1A). As expected, the liver was the unique organ exhibiting a strong reduction of *Napepld* mRNA expression confirming the liver specificity of the model. In accordance with this data, the deletion was also observed by in situ hybridization performed on liver slices of both genotypes since the *Napepld*-specific probe was not detected in *Napepld*^∆Hep^ hepatocytes (Figure S1A).

Next, we measured the NAEs produced by NAPE-PLD in the liver of WT and *Napepld*^∆Hep^ mice (Figure 1B). The levels of OEA and LEA were significantly lower, as mirrored by the 20% reduction, in *Napepld*^∆Hep^ livers compared to WT whereas no change was reported regarding AEA and PEA levels. These data support the existence of alternative pathways, possibly tissue- and cell-specific for NAE biosynthesis. This alternative pathway has been largely described for AEA biosynthesis, which is also consistent with our previous studies [15,16,21,33,34,35]. Surprisingly, almost all the lipid congeners related to the eCBome (i.e., acylglycerols) were affected in the liver of *Napepld*^∆Hep^ mice, suggesting that NAPE-PLD could, albeit indirectly, control the biosynthesis of a larger group of bioactive lipids in the liver than initially thought (Figure 1C and Figure S1B). The altered lipid profile could not be explained by a major impact on the other biosynthetic and degrading enzymes for these mediators because the monoacylglycerol lipase (*Mgll)* was the only enzyme significantly altered at the mRNA level, as reflected by a 25% decreased expression, in *Napepld*^∆Hep^ livers as compared to WT (Figure 1D).

To further validate the specific deletion of NAPE-PLD in hepatocytes, we separated these cells from the hepatic non-parenchymal cells (NPC). Of interest, the purity of both fractions was measured by various markers: the NPC fraction was assessed by an increased mRNA expression of *F4/80*, *Cd31*, *Acta2* and *Ck19,* which are specific to Kupffer cells, sinusoidal endothelial cells, stellate cells and cholangiocytes respectively whereas a higher expression of *Hnf4a* was unique to the hepatocyte fraction (Figure S1C) [36,37,38,39]. Our data showed that the expression of *Napepld* was markedly decreased in the hepatocyte enriched fraction from *Napepld*^∆Hep^ mice, while the NPC were not significantly affected (Figure 1F).

Altogether, these data confirm the invalidation of the *Napepld* gene in hepatocytes and highlight a new role of NAPE-PLD in the regulation of liver lipid metabolism.

### 3.2. Napepld^∆Hep^ Mice Develop a High-Fat Diet-Like Phenotype upon Normal Diet

**Figure 2:**
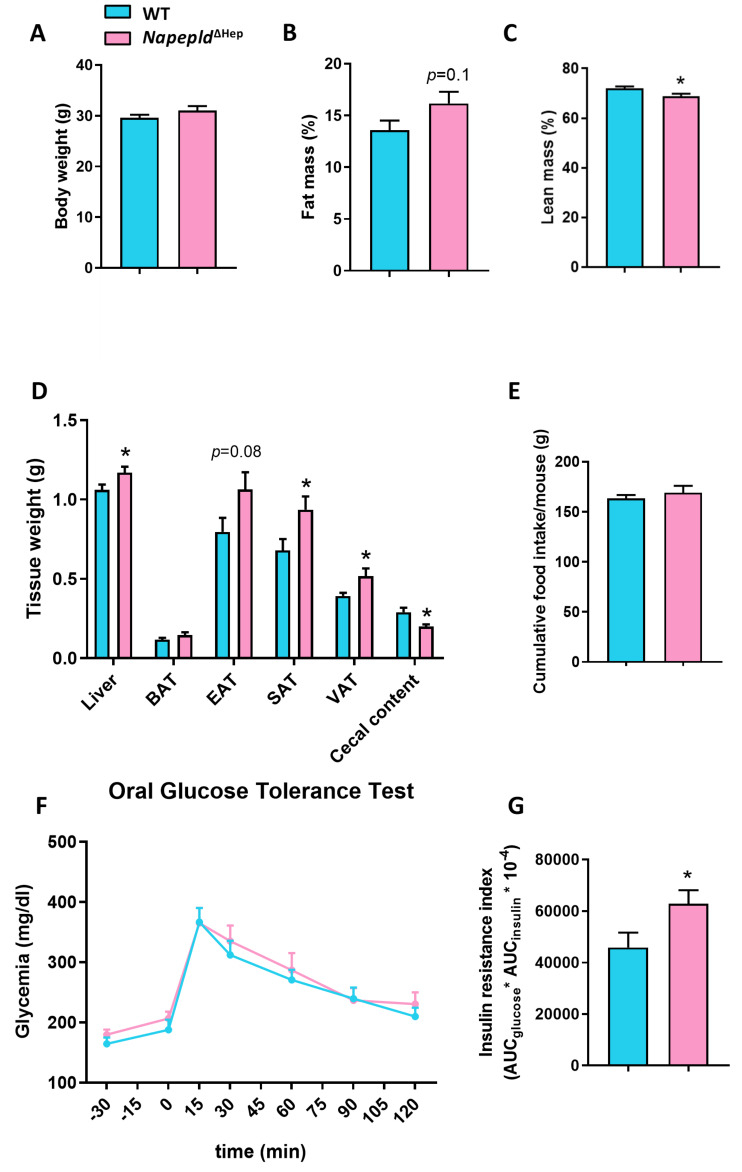
*Napepld*^∆Hep^ mice develop a high-fat diet-like phenotype upon normal diet. (**A**) Body weight (g), (**B**) fat mass (%) and (**C**) lean mass (%) after a 7 weeks period on normal diet. (**D**) Weight (g) of different tissues: liver, brown adipose tissue (BAT), epididymal adipose tissue (EAT), subcutaneous adipose tissue (SAT), visceral adipose tissue (VAT) and cecal content. (**E**) Cumulative food intake per mouse (g) after a 7 weeks period. (**F**) Plasma glucose profile (mg/dl) measured between 30 min before and 120 min after oral glucose loading (**G**) Insulin resistance index determined by multiplying the area under the curve (AUC) of blood glucose by the AUC of insulin. Blue: WT ND mice. Pink: *Napepld*^∆Hep^ ND mice (*n* = 8–10). Data are presented as the mean ± s.e.m. *and ** indicate a significant difference versus WT ND (Respectively *p* < 0.05 and *p* < 0.01) according to the *t*-test.

To assess whether the *Napepld* deletion in hepatocytes affects the whole-body metabolism, we followed a cohort of WT and *Napepld*^∆Hep^ mice fed a control diet for 7 weeks. Strikingly, although the body weight of the two genotypes was equivalent at the end of the experiment, nuclear magnetic resonance (NMR) revealed that *Napepld*^∆Hep^ mice tended to have a higher percentage of fat mass and a significantly lower percentage of lean mass compared to WT mice (Figure 2A–C). The fat mass accumulation was reflected by increased adipose tissue weights in *Napepld*^∆Hep^ mice, obtained after precise dissection. The liver weight of *Napepld*^∆Hep^ mice was also higher compared to WT mice, while a significant reduction of the cecal content was observed in *Napepld*^∆Hep^ mice (Figure 2D). All the aforementioned measures observed in *Napepld*^∆Hep^ mice are usually reported in the animal model of diet-induced obesity and this effect could not be attributed to an increase in food intake (Figure 2E). Therefore, we evaluated the impact of hepatocyte *Napepld* deletion on whole-body glucose metabolism by performing an oral glucose tolerance test (OGTT). The glycemia of WT and *Napepld*^∆Hep^ mice was similar during the test (Figure 2F). However, *Napepld*^∆Hep^ mice exhibited a higher insulin resistance index (Figure 2G), mainly explained by elevated plasma insulin levels (Figure S2). 

Overall, *Napepld*^∆Hep^ mice display a high-fat diet-like phenotype without developing glucose intolerance. 

### 3.3. Napepld^∆Hep^ Mice Are More Sensitive to Liver Lipid Accumulation

**Figure 3:**
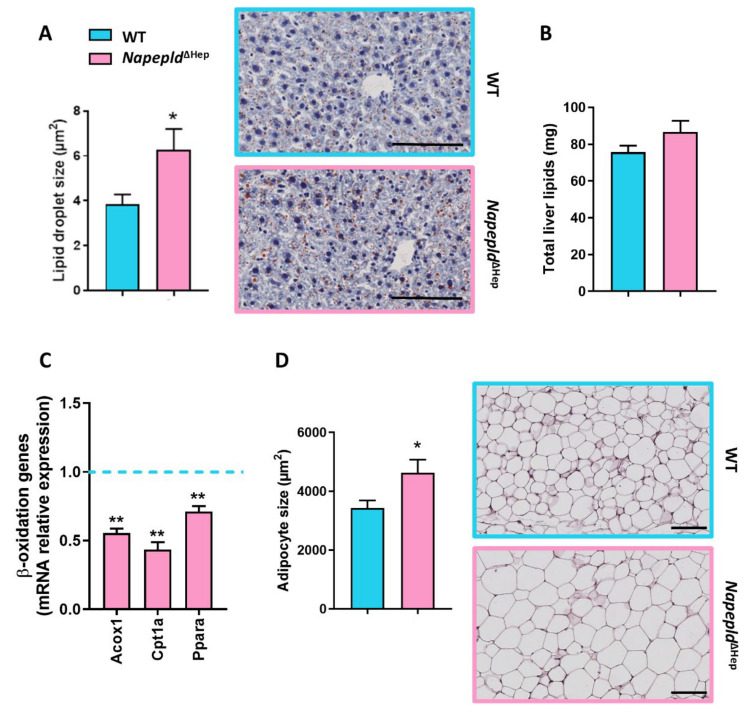
*Napepld*^∆Hep^ mice are more sensitive to liver lipid accumulation. (**A**) Representative liver oil red O staining (scale bar: 100 µm) and average lipid droplet size (μm²). (**B**) Liver lipid content measured by gravimetry (mg). (**C**) mRNA expression of genes involved in β-oxidation measured by real-time qPCR in the liver. (**D**) Representative hematoxylin and eosin-stained pictures of SAT deposits (scale bar: 100 µm) and mean adipocyte size (μm²). Blue: WT ND mice. Pink: *Napepld*^∆Hep^ ND mice (*n* = 8–10). Data are presented as the mean ± s.e.m. *and ** indicate a significant difference versus WT ND (Respectively *p* < 0.05 and *p* < 0.01) according to the *t*-test.

Since *Napepld*^∆Hep^ mice displayed an altered liver lipid metabolism as well as a higher liver weight, we wondered whether this latter was due to an accumulation of liver lipids. To address this question, we first performed histology analysis by staining liver slices of WT and *Napepld*^∆Hep^ mice with oil red O dye, which highlights lipid droplet in red. Although the global liver morphology was similar between both genotypes, the lipid droplet size was larger in *Napepld*^∆Hep^ liver compared to WT (Figure 3A). In parallel, gravimetric and biochemical measurements were conducted to quantify liver lipid content in WT and *Napepld*^∆Hep^ mice. In accordance with the 10% increased of liver mass, the results, albeit not significant, showed a slight elevation in total liver lipid (15%) and triglyceride (11%) levels in *Napepld*^∆Hep^ liver (Figure 3B and Figure S3A). To gain a better insight into the origins of the higher liver lipid accumulation, we investigated the enzymes involved in the β-oxidation pathway including peroxisomal acyl-coenzyme A oxidase 1 (*Acox1*), carnitine O-palmitoyltransferase 1α (*Cpt1a*) and peroxisome proliferator-activated receptorα (*Ppara*; Figure 3C). Strikingly, all studied genes were downregulated at the mRNA level indicating that the absence of *Napepld* in hepatocytes could contribute to this phenomenon. Besides, we also explored the adipose tissue metabolism given that this organ plays a pivotal role on lipid storage and release. By carrying out hematoxylin and eosin staining on SAT deposits, we discovered that the size of adipocytes was larger in *Napepld*^∆Hep^ mice strengthening the high-fat diet-like phenotype exhibited by these mutant mice (Figure 3D). Additionally, because insulin resistance can be linked with a higher release of circulating free fatty acids promoted by the lipolysis and that *Napepld*^∆Hep^ mice had a higher insulin resistance index, we quantified plasma lipid levels. We discovered that plasma triglyceride level tended to increase (*p* = 0.05) whereas plasma non-esterified fatty acids (NEFA) and cholesterol levels were unchanged in both groups (Figure S3B–D).

All the aforementioned results indicate that deletion of NAPE-PLD in hepatocytes contributes to liver lipid accumulation by affecting β-oxidation pathway rather than lipolysis.

### 3.4. Hepatocyte Napepld Deletion Modifies Liver Bioactive Lipid Metabolism

**Figure 4:**
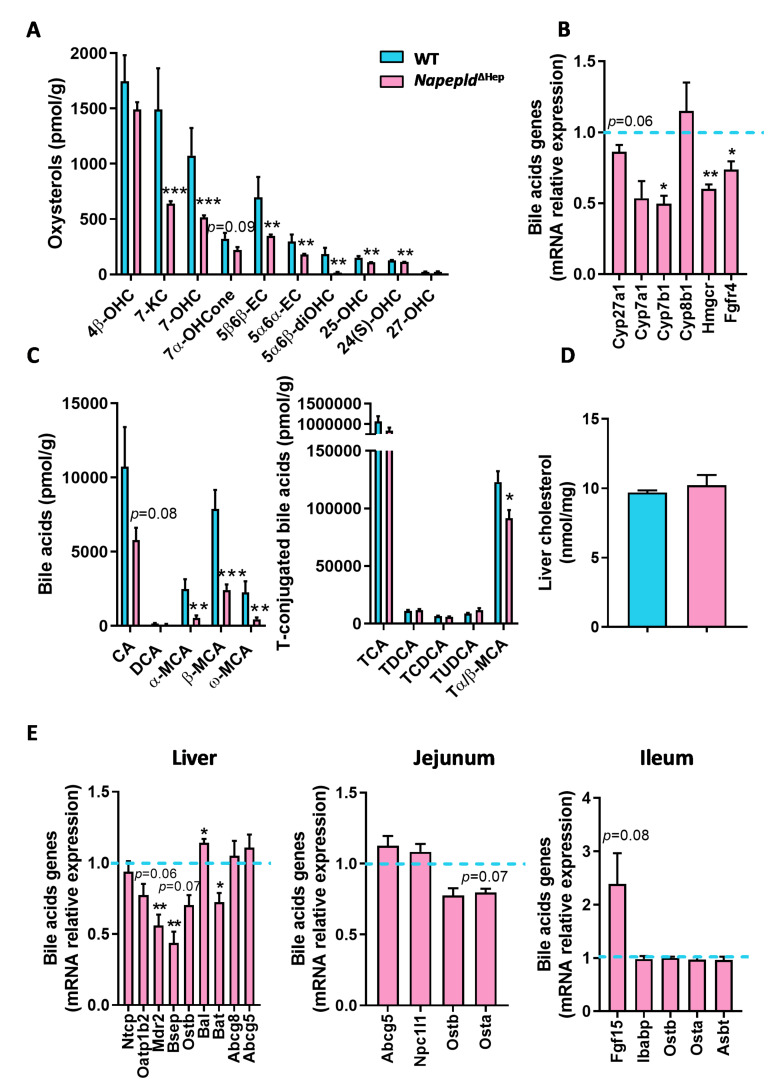
Hepatocyte *Napepld* deletion modifies liver bioactive lipid metabolism. (**A**) Liver oxysterol concentration (pmol/g). (**B**) Key markers of bile acid metabolism measured by real-time qPCR in the liver. (**C**) Liver bile acid concentration (pmol/g). (**D**) Liver cholesterol concentration (nmol/mg). (**E**) Markers of the enterohepatic feedback loop involved in bile acid metabolism measured by real-time qPCR in the liver, jejunum and ileum. Blue: WT ND mice. Pink: *Napepld*^∆Hep^ ND mice (*n* = 8–10). Data are presented as the mean ± s.e.m. *, ** and *** indicate a significant difference versus WT ND (Respectively *p* < 0.05, *p* < 0.01 and *p* < 0.001) according to the *t*-test. Lipid abbreviations: 24(S)-OHC, 24(S)-hydroxycholesterol; 25-OHC, 25-hydroxycholesterol; 27-OHC, 27-hydroxycholesterol; 4β-OHC, 4β-hydroxycholesterol; 5α6α-EC, 5α,6α-epoxycholesterol; 5α6β-diOHC, 5α,6β-dihydroxycholesterol; 5β6β-EC, 5β,6β-epoxycholesterol; 7-KC, 7-ketocholesterol; 7-OHC, 7-hydroxycholesterol; 7α-OHCone, 7α-hydroxycholestenone; CA, Cholic acid; CDCA, Chenodeoxycholic acid; DCA, Deoxycholic acid; (α, β or ω)-MCA, (α, β or ω)-Muricholic acid.

Our first liver lipidomic analysis regarding NAEs and eCBome mediators comparing WT and *Napepld*^∆Hep^ mice revealed that NAPE-PLD deletion impacts lipid regulation beyond NAEs synthesis. To investigate the effect of NAPE-PLD deletion on additional lipid mediators in the liver, we quantified two important liver lipid families derived from cholesterol oxidation, namely the bile acids and their precursors, the oxysterols. Surprisingly, almost all bioactive lipids belonging to both families were strongly decreased in *Napepld*^∆Hep^ mice in comparison to WT (Figure 4A,C). To better characterize this altered lipid profile, we studied the gene expression of enzymes responsible for the synthesis of cholesterol and bile acid metabolism. We found a significant downregulation of *Cyp7b1* and *Hmgcr* mRNA and a tendency for decreased *Cyp27a1* mRNA (*p* = 0.06; Figure 4B). This finding is in line with the reduced concentrations of these bioactive lipids in the liver of *Napepld*^∆Hep^ mice. We also explored the enterohepatic feedback loop of bile acid metabolism by examining the mRNA expression of receptors and transporters related to the recirculation of bile acids to the liver (Figure 4E). After their synthesis in hepatocytes and prior to secretion into the biliary canaliculi, bile acids must be conjugated with taurine in mice or glycine in humans. Interestingly, the enzymes involved in bile acid conjugation, bile acid CoA ligase (*Bal*) and bile acid CoA:amino acid N-acyltransferase (*Bat*), were already altered in this first step in *Napepld*^∆Hep^ mice. Following conjugation, bile acids cannot diffuse across membranes and need specific transporters to go through hepatocyte membrane. As such, the organic solute transporter (OST)β mediates bile acid efflux and the bile salt export protein (BSEP) allows them to reach biliary canaliculi. In this study, we found that *Ostb* tended to be reduced (*p* = 0.07) while *Bsep* was significantly downregulated *in Napepld*^∆Hep^ mice suggesting a decreased secretion of bile salts. Of interest, the bile is also composed of phospholipids and cholesterol, which are transported from hepatocytes to the gallbladder by respectively multidrug resistance protein 2 (MDR2) and ATP-binding cassette sub-family G member (ABCG)5 and ABCG8. *Mdr2* mRNA expression was significantly decreased in *Napepld*^∆Hep^ livers whereas cholesterol transporters were reported unchanged. In addition, transporters in charge of the reabsorption of bile acids in hepatocytes such as the sodium (Na^+^)-taurocholate cotransporting polypeptide (NTCP) and the organic anion transporter (OATP)1B2 were also investigated. Although *Ntcp* mRNA expression was similar in both groups, *Oatp1b2* tended to be reduced in *Napepld*^∆Hep^ mice (*p* = 0.06). In the jejunum and ileum, however, we found no significant difference in mRNA level of bile acid transporters such as the apical sodium-dependent transporter (ASBT) or OSTα/OSTβ that would have indicated a change in bile acid reabsorption. Besides, the expression of intestinal bile acid-binding protein (*Ibabp*) that facilitates the travel of bile acids from the apical to the basolateral membrane of enterocytes was not different either between groups. Of note, the enterohepatic feedback loop is also driven by the stimulation of the endocrine hormone fibroblast growth factor (FGF)-15. This latter is induced by the activation of farnesoid receptor X (FXR) by specific bile acids in the enterocyte and then secreted into the portal circulation to repress bile acid synthesis by inhibiting cytochrome P450 enzyme (CYP)7A1 and (CYP)8B1 via fibroblast growth factor receptor (FGFR)4 receptor expressed in the liver. We found that *Fgf15* expression doubled in the ileum of *Napepld*^∆Hep^ mice as compared to WT (*p* = 0.08) whereas *Fgfr4* was downregulated. Although the increased level of *Fgf15* could contribute mildly to the slight decrease in *Cyp7a1* expression, no significant repression was reported regarding *Cyp7a1* and *Cyp8b1* mRNA expressions in the liver of *Napepld*^∆Hep^ mice. This suggests that the enterohepatic feedback loop is functional in enterocytes but not properly active in the liver. Finally, the deletion of hepatocyte *Napepld* did not change total liver cholesterol content (Figure 4D) nor the expression of cholesterol transporters measured both in the liver (*Abcg5* and *Abcg8*) and in the intestine (*Abcg5* and *Npc1l1).* Thereby, it indicates that hepatocyte NAPE-PLD influences bile acid and oxysterol production without affecting cholesterol secretion and absorption.

Taken together, these findings demonstrate a strong link between hepatic *Napepld* and the regulation of cholesterol-derived lipid metabolism.

### 3.5. Deletion of Napepld Partially Accentuates the Obese Phenotype Induced by a High-Fat Diet

**Figure 5:**
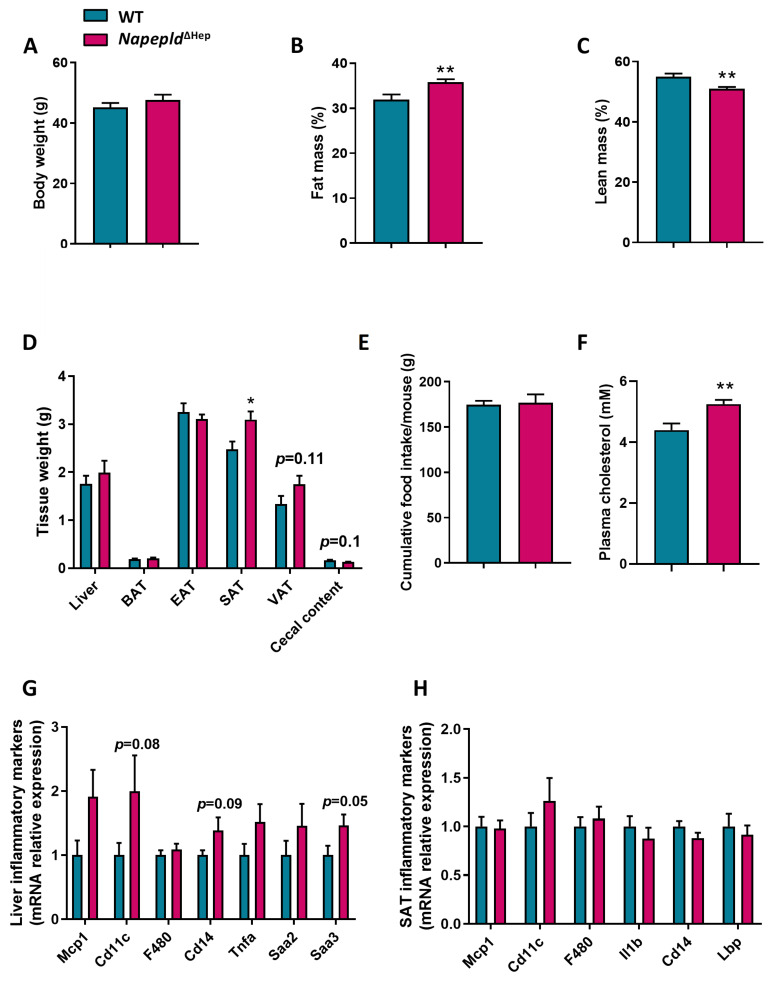
Deletion of *Napepld* partially accentuates the obese phenotype induced by a high-fat diet. (**A**) Body weight (g) after an 8 weeks period. (**B**) Fat mass (%) over an 8 weeks period. (**C**) Lean mass (%) after an 8 weeks period. (**D**) Weight (g) of different tissues: liver, brown adipose tissue (BAT), epididymal adipose tissue (EAT), subcutaneous adipose tissue (SAT), visceral adipose tissue (VAT) and cecal content. (**E**) Cumulative food intake per mouse (g) after an 8 weeks period. (**F**) Plasma cholesterol concentration (mM). (**G**) Liver and (**H**) SAT inflammatory markers measured by real-time qPCR. Dark blue: WT HFD mice. Dark pink: *Napepld*^∆Hep^ HFD mice (*n* = 11). Data are presented as the mean ± s.e.m. * and ** indicate a significant difference versus WT HFD (Respectively *p* < 0.05 and *p* < 0.01) according to the *t*-test (except for (B) that is according to a two-way ANOVA followed by a Tukey post hoc test).

Given that, in the absence of *Napepld* in hepatocytes, the mice fed control diet developed a high-fat diet-like phenotype, we wondered whether challenging the mice with a high-fat diet (HFD) would worsen the phenotype. After 8 weeks of HFD, *Napepld*^∆Hep^ mice had a higher percentage of fat mass and a decreased percentage of lean mass compared to WT. This was independent of food intake or body weight (Figure 5A–C,E). Subcutaneous adipose tissue (SAT) was significantly increased and visceral adipose tissue (VAT) tended to be larger (*p* = 0.1) in *Napepld*^∆Hep^ mice. Similarly to what observed upon the normal diet, in this experiment the cecal content also tended to be reduced in *Napepld*^∆Hep^ mice as compared to WT mice exposed to HFD (Figure 5D). Moreover, biochemical analyses showed an elevated concentration of plasma cholesterol in *Napepld*^∆Hep^ mice whereas no change was reported for plasma triglycerides (Figure 5F and Figure S4F).

Although, *Napepld*^∆Hep^ mice exhibited an exacerbated fat mass accumulation, they did not develop a worsened glucose intolerance nor higher plasma insulin level compared to WT during the OGTT suggesting that the potential effect of NAPE-PLD on insulin level might be hidden by the diet itself, which could be the main driver (Figure S4A–E). However, the inflammatory tone in the liver of *Napepld*^∆Hep^ mice tended to be higher as reflected by RT-qPCR measurements of inflammatory markers such as cluster of differentiation 11c (*Cd11c; p* = 0.08) that reflects activated macrophages, cluster of differentiation 14 (*Cd14; p* = 0.09) that contributes to endotoxin-induced inflammation or serum amyloid A3 (*Saa3; p* = 0.05) which encodes an acute phase protein that increases upon inflammation (Figure 5G). Of interest, this trend was not observed in adipose tissue suggesting that hepatocyte NAPE-PLD exerts a substantial function locally rather than distally regarding the inflammatory response (Figure 5H).

Collectively, the results show that following a nutritional stress, *Napepld*^∆Hep^ mice store a greater amount of fat and seem predisposed to develop high-fat diet-induced inflammation.

### 3.6. Napepld^∆Hep^ Mice are More Sensitive to Inflammation

**Figure 6:**
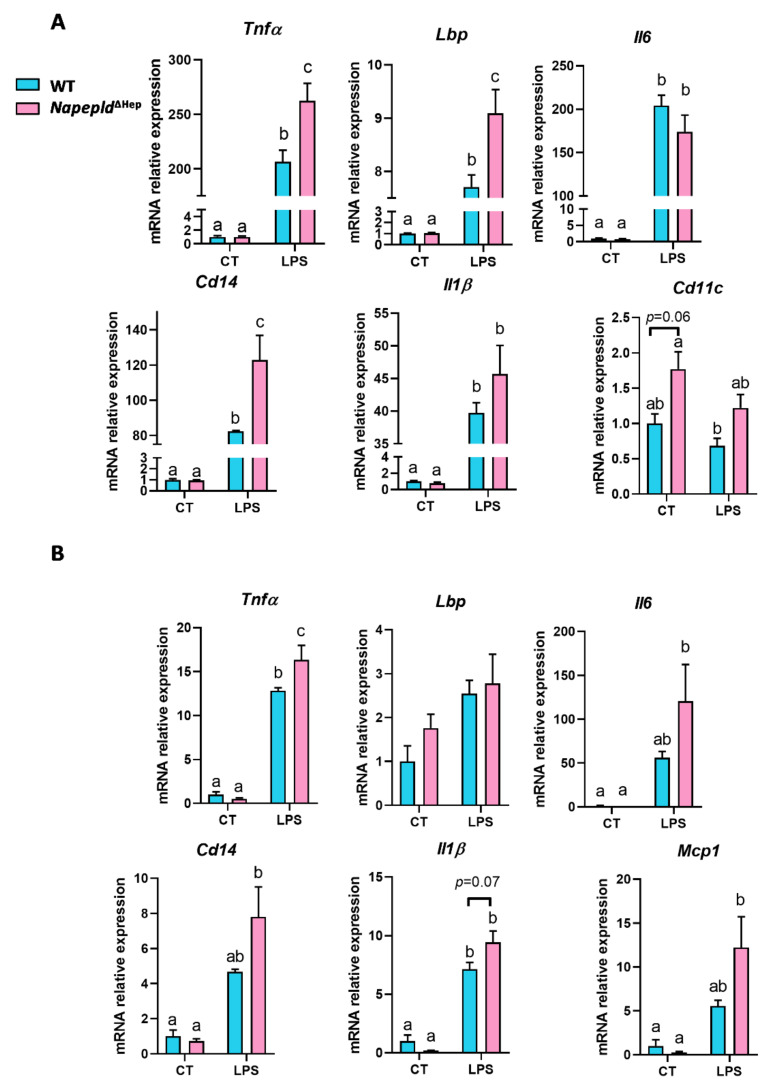
*Napepld*^∆Hep^ mice are more sensitive to inflammation. mRNA expression of inflammatory markers measured by real-time qPCR in WT and *Napepld*^∆Hep^ mice injected with either saline solution (control (CT)) or LPS (**A**) in the liver and (**B**) in the subcutaneous adipose tissue (SAT). Blue: WT ND mice. Pink: *Napepld*^∆Hep^ ND mice. Data are presented as the mean ± s.e.m. (*n* = 4–6). Data with different superscript letters are significantly different (*p* < 0.05) according to a two-way ANOVA followed by a Tukey post hoc test.

To better evaluate the involvement of hepatocyte *Napepld* in the inflammatory response, we injected WT and *Napepld*^∆Hep^ mice with lipopolysaccharides (LPS), a known potent proinflammatory compound. LPS challenge induced an acute inflammation in the liver of both WT and *Napepld*^∆Hep^ mice (Figure 6A), but the inflammatory tone was worsened in the liver of *Napepld*^∆Hep^ mice as reflected by the increased mRNA expression of inflammatory markers. Even though, proinflammatory cytokines interleukin-1β (*Il1β*) and interleukin-6 (*Il6*) did not reach the significance, tumor necrosis factor alpha (*Tnfα*) was significantly upregulated in *Napepld*^∆Hep^ livers. In line with this, gene expression of proteins contributing to the inflammatory response triggered by LPS such as LPS-binding protein (*Lbp*) and *Cd14* were both significantly increased in *Napepld*^∆Hep^ mice. Interestingly, the expression of *Cd11c*, a marker of activated macrophages, tended to be more elevated in *Napepld*^∆Hep^ mice already in basal conditions (*p* = 0.06) compared to WT mice. This might explain the predisposition of these mice to develop a higher inflammatory tone when challenged by a chronic or acute inflammation.

Additionally, we examined the inflammation in the subcutaneous adipose tissue to investigate whether hepatocyte *Napepld* also affects inflammatory response in another peripheral organ involved in obesity. The magnitude of the increase in the LPS-induced inflammation markers was lower in the adipose tissue as compared to the liver. Although only *Tnfα* expression was significantly higher in *Napepld*^∆Hep^ mice, a consistent trend was observed for the other inflammatory markers when compared to WT mice (Figure 6B).

These data suggest that the marked reduction in the different bioactive lipids that regulate inflammation, caused by the deletion of *Napepld* in the liver, affects primarily the immune response in the liver itself and to a lesser extent that in the periphery.

## 4. Discussion

The bioactive lipids belonging to the eCBome are involved in numerous metabolic functions, including lipid and glucose metabolism, energy homeostasis and regulation of inflammatory tone [2,40]. In this study, we discovered that hepatocyte NAPE-PLD plays a major role in most of these physiological processes.

Regarding the direct targets of NAPE-PLD, the NAEs [35,41,42], we found a significant decrease in OEA and LEA concentrations in the liver of *Napepld*^∆Hep^ mice, whereas AEA and PEA levels remained unchanged. The lack of reduction of PEA and AEA levels could not be explained by a downregulation of the enzyme in charge of their degradation since the expression of both fatty acid amide hydrolase (*Faah)* and *N*-acylethanolamine acid amide hydrolase (*Naaa*) was unaltered in *Napepld*^∆Hep^ mice [43,44]. However, alternative biosynthetic pathways have already been demonstrated for some NAEs and especially for AEA [15,16,21,33,34,35]. Even tough NAPE-PLD is the main enzyme producing NAEs, we cannot rule out that the serine hydrolase α/β -hydrolase 4 (ABHD4) and the glycerophosphodiesterase 1 (GDE1) may also partially contribute to their synthesis [35]. Finally, among the different roles of PEA, it is well-documented that this NAE displays anti-inflammatory properties and is produced by immune cells [45,46,47,48,49,50]. As we observed that NPC also expressed *Napepld*, we could not exclude that the absence of a decreased concentration of this mediator, or even AEA, in the tissue was due to compensatory mechanisms from cells unaffected by the deletion construct, possibly immune cells (i.e., Kupffer cells).

More surprisingly, NAPE-PLD deletion led to a robust and consistent reduction of almost all other eCBome mediators investigated here, suggesting that hepatocyte NAPE-PLD controls bioactive liver lipid metabolism that goes beyond NAEs production, which is the only reported function of NAPE-PLD so far [42]. Our data showed that the absence of NAPE-PLD in hepatocytes did not alter the expression of other key enzymes of the eCBome except one, the monoacylglycerol lipase (*Mgll*), which was downregulated. Since this enzyme is an endocannabinoid-glycerol lipase, as well as *Abhd6* that tended to be decreased (*p* = 0.08), one would have expected an accumulation of monoacylglycerols into *Napepld*^∆Hep^ livers. Strikingly, the results indicated the opposite effect. As other endocannabinoid-glycerol lipases have recently been discovered in human leukocytes [51], we cannot rule out that they do not take part into the degradation of monoacylglycerols in the mouse liver. This assumption deserves however further investigations. Interestingly, *Mgll*, aside from degrading monoacylglycerols is also involved in the hydrolysis of triglycerides to fatty acid and glycerol [35,52]. As *Napepld*^∆Hep^ mice did not accumulate monoacylglycerols in the liver, we hypothesized that the decreased *Mgll* mRNA expression may restrict glycerol availability and contribute to the reduction of the concentrations of glycerol-associated lipids. This was also reflected by the increased levels of arachidonic acid (AA) in *Napepld*^∆Hep^ mice whereas its glycerol-conjugated forms, 1/2-AG, were strongly decreased.

The hypothesis that NAPE-PLD acts as a hub controlling, albeit indirectly, the levels of a large range of liver lipids was further confirmed by the lipidomic analysis of two essential liver bioactive lipid families, namely the bile acids and the oxysterols. Strikingly, although most of the bile acids and the oxysterols were strongly reduced in *Napepld*^∆Hep^ mice, the level of cholesterol, which is the precursor of these lipids was not affected by the lack of *Napepld* in hepatocytes. Besides, the expression of cholesterol transporters measured in the liver and in the intestine (*Abcg5/8* and *Npc1l1*) was similar between *Napepld*^∆Hep^ mice and WT. However, several enzymes involved in bile acids synthesis were significantly, or tended to be, downregulated in *Napepld*^∆Hep^ mice. Interestingly, these enzymes (*Cyp27a1* and *Cyp7b1*) are involved in the alternative pathway of bile acids (as opposed to the classic pathway) and produce muricholic acids in rodents [53,54]. This is in line with the marked reduction in muricholic acids (i.e., α-MCA, β-MCA, ω-MCA and to a lower extent Tα/β-MCA) observed in *Napepld*^∆Hep^ mice.

The enterohepatic feedback loop also contributes to the reduction of bile acid synthesis [55,56]. By investigating this pathway we discovered that the main transporters of bile acids regulating their reabsorption in the ileum (*Osta/b* and *Asbt*) [57] were not altered, whereas the majority of the liver-specific transporters were reduced in *Napepld*^∆Hep^ mice (*Oatp1b2*, *Bsep* and *Ostb*). This negative feedback loop also involves a bile acid–FXR–FGF15 axis, in which specific bile acids bind to FXR in the intestine to induce the secretion of FGF15 into the portal circulation. FGF15 then binds to the heterodimeric FGFR4/βKlotho receptor and inhibits CYP7A1 and CYP8B1 expression in hepatocytes thereby limiting de novo synthesis of bile acids [58,59,60]. In *Napepld*^∆Hep^ mice, *Fgf15* was strongly expressed in the enterocyte, confirming the absorption of bile acids in the intestine. Yet, we did not see a significant decrease in *Cyp7a1* and *Cyp8b1* mRNA expression in the liver of *Napepld*^∆Hep^ mice, possibly due to a downregulation of the intermediate, *Fgfr4*. Of interest, the elevated *Fgf15* expression might be explained by a significant decrease in a well-characterized FXR antagonist, Tα/βMCA [61]. In comparison, FXR agonists such as TCDCA, TCA and TDCA were not significantly reduced. All the aforementioned results suggest that the enterohepatic loop was not altered in the intestine while it was impacted in the liver. Therefore, we may conclude that the reduced production of bile acids was not likely due to FGF15/FGFR4 signaling pathway but rather to an altered regulation in the liver itself. Since cholesterol levels in WT and *Napepld*^∆Hep^ mice were similar but the cholesterol-derived metabolites were deeply modified in *Napepld*^∆Hep^ livers, we hypothesized that hepatic NAPE-PLD modulates the cascade participating to cholesterol oxidation and specifically the alternative pathway of bile acid synthesis rather than the classic pathway given that this latter was only slightly affected. This possibility, and the underlying molecular mechanisms, will need to be explored in future studies.

It is also worth mentioning that secondary bile acids (i.e., LCA and DCA) are potential structural cofactors regulating NAPE-PLD activity [13,14]. Interestingly, in our study, by deleting NAPE-PLD we discovered that *Napepld*^∆Hep^ mice were characterized by a specific change in bile acid profile. Hence, given that bile acid metabolism is highly affected in *Napepld*^∆Hep^ mice and that different data have linked bile acids with the regulation of NAPE-PLD activity [13,14], we speculated on the existence of a mutual regulation between bile acids and NAPE-PLD activity. Nonetheless, this assumption deserves further investigations.

One of the remarkable outcomes of this study is that a targeted deletion for *Napepld* in hepatocytes is sufficient to induce a high-fat diet-like phenotype. Indeed, we showed that *Napepld*^∆Hep^ mice were more sensitive to accumulate liver lipids as mirrored by the increased liver weight and the larger vacuolar lipid droplets highlighted by oil-red O staining. We indicated that this phenomenon was likely due to a decreased expression of genes involved in the β-oxidation process rather than an increase in circulating fatty acid level. Indeed, since *Napepld*^∆Hep^ mice also accumulated fat mass in various adipose tissues, had larger adipocytes and exhibited a higher insulin resistance index compared to WT mice, the lipolysis pathway could have been promoted. Nevertheless, the plasma NEFA level was similar in both genotypes suggesting that adipose tissue was still responsive to insulin action. This obesity-prone phenotype in *Napepld*^∆Hep^ mice existed already in basal conditions under a normal chow diet and was partially accentuated upon HFD exposure. These observations corroborate a central role of NAPE-PLD in lipid metabolism as a higher accumulation of fat mass has also been reported in mice lacking *Napepld* in either the adipose tissue or the intestinal epithelial cells [21,22]. Interestingly, in this present study, we could hypothesize that this higher body fat content might be linked to the decreased levels of monoacylglycerols in *Napepld*^∆Hep^ mice since mice globally deleted for *Mgll* accumulate monoacylglycerols and display a leaner phenotype [62]. However, the exact molecular mechanisms behind this connection remain to be elucidated.

Although glucose metabolism is also regulated by the endocannabinoid system [21,40,63,64], the liver glucose metabolism was not affected by the absence of *Napepld* in hepatocytes, neither under basal (i.e., control diet) nor under pathological conditions (i.e., diet-induced obesity). This absence of hyperglycemia in *Napepld*^∆Hep^ mice indicated that these mice were not yet strongly resistant to insulin despite their increased insulin resistance index under control diet. Since the timeline of events regarding hyperinsulinemia and insulin resistance is still under debates, we proposed that the hyperinsulinemia displayed by *Napepld*^∆Hep^ mice would be the cause of a future insulin resistance [65]. This suggests that *Napepld*^∆Hep^ mice were at the early stage of a diabetic disorder when exposed to a normal diet for seven weeks. Given that glucose metabolism and bile acids are intertwined, we speculated that the alteration of bile acids might be responsible of hyperinsulinemia. For instance, Jiang and colleagues demonstrated an improvement of glucose metabolism and a reduction of plasma insulin level in mice treated with glycine-β-MCA [66]. Interestingly, MCA were strongly reduced in *Napepld*^∆Hep^ mice. Nonetheless, the relationship between glucose metabolism and these bioactive lipids are not that clear and further investigations are needed in that field to fully identify the molecular interconnections [67]. Under HFD, *Napepld*^∆Hep^ mice and WT exhibited a similar level of insulin. This could be explained by the fact that HFD per se induces hyperinsulinemia and hyperglycemia suggesting that this factor is the major driver contributing to this phenotype and might hide the potential effect caused by *Napepld* deletion. Of note, intestinal *Napepld*-deleted mice do not develop glucose intolerance either unlike mice deleted for *Napepld* in the adipose tissue [21,22], reinforcing our hypothesis that NAPE-PLD plays an organ-dependent role in regulating lipid and glucose metabolism. These various roles might be explained with the fact that the enzyme controls the concentrations of different lipid mediators in specific cells: different NAEs in hepatocytes, adipocytes and epithelial intestinal cells as well as monoacylglycerols, bile acids and oxysterols in hepatocytes. These bioactive lipids display different or even opposite effects, mediated by different receptors (i.e., CB1, GPR119, GPR55, FXR and LXR), on lipid and glucose metabolism [56,68,69].

In addition to excessive fat mass, obesity is also linked to a low-grade inflammation [70]. Interestingly, we reported that when exposed to a nutritional stress such as a high-fat diet, *Napepld*^∆Hep^ mice tended to be more sensitive to liver inflammation compared to WT mice. To further explore this phenomenon, we challenged the mice with an acute exposure to LPS. We found that, following this treatment, *Napepld*^∆Hep^ mice exhibited a worsened inflammatory response in the liver. One explanation for this predisposition to inflammation may be found in the altered liver lipid profile of the *Napepld*^∆Hep^ mice. Indeed, AA, a polyunsaturated fatty acid, accumulated in the liver of *Napepld*^∆Hep^ mice. AA may come from the degradation of endocannabinoids such as AEA or 2-AG and is a precursor of prostaglandins, leukotrienes or lipoxins, which are involved in the inflammatory response [71,72,73,74]. Besides, eCBome mediators as well as oxysterols and bile acids regulate inflammation [69,73,75,76]. The relationship between immunity, inflammation, oxysterols and bile acids has also been described in our lab since we recently demonstrated that deletion of hepatocyte myeloid differentiation primary response gene 88 (MyD88) led to a profound alteration of bile acid and oxysterol profiles, which contributed to the increased inflammation sensitivity displayed by the mutant mice [28,77]. Hence, we may not exclude that the reduced levels of these molecules detected here in *Napepld*^∆Hep^ mice also participated to the observed sensitivity. Likewise, the decrease, found in these mice, in the hepatic levels of OEA, previously observed also in obese Zucker rats [78], in view of the PPARα agonist action of this NAE [72], may underlie both the excessive hepatic lipid accumulation and inflammation that are typical of both animal models. However, as both pro- and anti-inflammatory lipids were decreased in *Napepld*^∆Hep^ mouse livers, understanding their role in this context, particularly in view of their different relative potencies, would be rather complicated at this stage, thus calling for further research. Finally, it is important to note that the higher inflammatory tone was not observed in the adipose tissue, suggesting that the involvement of hepatocyte NAPE-PLD is restricted to local regulation of inflammation.

In conclusion, by generating a mouse model harboring a liver specific deletion of NAPE-PLD, we discovered that hepatocyte NAPE-PLD orchestrates the synthesis of a large range of bioactive lipids in the liver. We also found that hepatocyte NAPE-PLD acts as a crucial player on energy homeostasis since *Napepld*^∆Hep^ mice are prone to develop an obese-like phenotype and are predisposed to inflammation. These findings contribute to the understanding of the physiological and pathological roles of hepatocyte NAPE-PLD, and, when combined with our previous studies, shed light on the broad contribution of this enzyme in the development and the progression of metabolic disorders. 

## Figures and Tables

**Table 1:** Primers used for real-time qPCR.

Gene	Protein	Forward Primer Sequence (5’-3’)	Reverse Primer Sequence (5’-3’)
*Abcb11*	BSEP	AGATACAACCGAAGGGGACA	TCAACTTCTTCCACAAGCACA
*Abcb4*	MDR2	GAGCCCGTGCTGTTCTCTAC	TCTGTTTCTGTCCCCCACTC
*Abcg5*	ABCG5	ACCTTACCCACGGTTCCTTT	ACGCATAATCACTGCCTGCT
*Abcg8*	ABCG8	CCGTCGTCAGATTTCCAATGA	GGCTTCCGACCCATGAATG
*Abhd6*	ABHD6	CTGTCCATAGTGGGGCAAGT	TCAGATGGGTAGTAAGCGGC
*Acox1*	ACOX1	CTATGGGATCAGCCAGAAAGG	AGTCAAAGGCATCCACCAAAG
*Acta2*	αSMA	GGCTGGAGAATTGGATCT	CCAGCAAAGGTCAGAGAAGG
*Adgre1*	F4/80	TGACAACCAGACGGCTTGTG	GCAGGCGAGGAAAAGATAGTGT
*Baat*	BAT	GCACAGGCTCATCAACAAGA	TAGAGCACACCACGTTCCTG
*Ccl2*	MCP1	GCAGTTAACGCCCCACTCA	TCCAGCCTACTCATTGGGATCA
*Cd14*	CD14	CCTGCCCTCTCCACCTTAGAC	TCAGTCCTCTCTCGCCCAAT
*Cpt1a*	CPT1α	AGACCGTGAGGAACTCAAACCTAT	TGAAGAGTCGCTCCCACT
*Cyp27a1*	CYP27A1	TCTGGCTACCTGCACTTCCT	GTGTGTTGGATGTCGTGTCC
*Cyp7a1*	CYP7A1	GGGATTGCTGTGGTAGTGAGC	GGTATGGAATCAACCCGTTGTC
*Cyp7b1*	CYP7B1	TAGGCATGACGATCCTGAAA	TCTCTGGTGAAGTGGACTGAAA
*Cyp8b1*	CYP8B1	GATCCGTCGCGGAGATAAGG	CGGGTTGAGGAACCGATCAT
*Daglb*	DAGLβ	CTCCACCAGCAACAAGACAA	GCAGTTCTCCACTTCTGCATC
*Faah*	FAAH	GTGAGGATTTGTTCCGCTTG	GGAGTGGGCATGGTGTAGTT
*Fabp6*	IBABP	CAAGGCTACCGTGAAGATGGA	CCCACGACCTCCGAAGTCT
*Fgf15*	FGF15	GAGGACCAAAACGAACGAAATT	ACGTCCTTGATGGCAATCG
*Fgfr4*	FGFR4	CTCGATCCGCTTTGGGAATTC	CAGGTCTGCCAAATCCTTGTC
*Hmgcr*	HMGCR	TGGTGGGACCAACCTTCTAC	GCCATCACAGTGCCACATAC
*Hnf4a*	HNF4α	AAGAGGTCCATGGTGTTTAAGG	ATCGAGGATGCGGATGGA
*Il1b*	IL1β	TCGCTCAGGGTCACAAGAAA	CATCAGAGGCAAGGAGGAAAAC
*Il6*	IL6	ACAAGTCGGAGGCTTAATTACACAT	TTGCCATTGCACAACTCTTTTC
*Itgax*	CD11c	ACGTCAGTACAAGGAGATGTTGGA	ATCCTATTGCAGAATGCTTCTTTACC
*Krt19*	CK19	AGCGTGATCAGCGGTTTTG	CCTGGTTCTGGCGCTCTATG
*Lbp*	LBP	GTCCTGGGAATCTGTCCTTG	CCGGTAACCTTGCTGTTGTT
*Mgll*	MGL	ATGGTCCTGATTTCACCTCTGGT	TCAACCTCCGACTTGTTCCGAGACA
*Naaa*	NAAA	ATTATGACCATTGGAAGCCTGCA	CGCTCATCACTGTAGTATAAATTGTGTAG
*Napepld*	NAPE-PLD	CTCCATCCCGAATGTGCT	AAGCCAGCCTCTCTCACTCC
*Npc1l1*	NPC1L1	GGCTCCATCTGGAGTAGCTG	ATCGCACTACCATCCAGGAC
*Oatp1b2*	OATP1B2	ATCCCGTGACTAATCCAACA	ACCAAACTGCTGCTCTATAAACT
*Pecam1*	CD31	GGAACGAGAGCCACAGAGAC	TGCACTGCCTTGACTGTCTT
*Ppara*	PPARα	CAACGGCGTCGAAGACAAA	TGACGGTCTCCACGGACAT
*Rpl19*	RPL19	GAAGGTCAAAGGGAATGTGTTCA	CCTTGTCTGCCTTCAGCTTGT
*Saa2*	SAA2	GGGGTCTGGGCTTCCTATCT	CCATTCTGAAACCCTTGTGG
*Saa3*	SAA3	CGCAGCACGAGCAGGAT	CCAGGATCAAGATGCAAAGAATG
*Slc10a1*	NTCP	GGACAAGGTGCCCTACAAAG	ACAGCCACAGAGAGGGAGAA
*Slc10a2*	ASBT	TGGGTTTCTTCCTGGCTAGACT	TGTTCTGCATTCCAGTTTCCAA
*Slc27a5*	BAL	TGTGTGTGAAGGAACCTGGA	ACCCGGACAACTTTGTGAAG
*Slc51a*	OSTα	TACAAGAACACCCTTTGCCC	CGAGGAATCCAGAGACCAAA
*Slc51b*	OSTβ	GTATTTTCGTGCAGAAGATGCG	TTTCTGTTTGCCAGGATGCTC
*Tnf*	TNFα	TCGAGTGACAAGCCTGTAGCC	TTGAGATCCATGCCGTTGG

**Table 2:** Lipids abbreviations.

NAEs and ECB-Related Molecules		Bile Acids	
**1-AG**	1-arachidonoylglycerol	CA	Cholic acid
**2-AG**	2-arachidonoylglycerol	CDCA	Chenodeoxycholic acid
**1-LG**	1-linoleoylglycerol	DCA	Deoxycholic acid
**2-LG**	2-linoleoylglycerol	(α, β or ω) MCA	(α, β or ω) Muricholic acid
**2-OG**	2-oleoylglycerol		
**1-PG**	1-palmitoylglycerol		
**2-PG**	2-palmitoylglycerol		
**AA**	Arachidonic acid		
**AEA**	*N*-arachidonoylethanolamine	**Oxysterols**	
**DHA**	Docosahexaenoic acid	24(*S*)-OHC	24(*S*)-hydroxycholesterol
**DHA-1-G**	DHA-1-glycerol	25-OHC	25-hydroxycholesterol
**DHA-2-G**	DHA-2-glycerol	27-OHC	27-hydroxycholesterol
**DHEA**	*N*-docosahexaenoylethanolamine	4β-OHC	4β-hydroxycholesterol
**DPA-1-G**	DPA-1-glycerol	5α6α-EC	5α,6α-epoxycholesterol
**DPA-2-G**	DPA-2-glycerol	5α6β-diOHC	5α,6β-dihydroxycholesterol
**DPA-ω3**	DPA-omega3	5β6β-EC	5β,6β-epoxycholesterol
**EPA**	Eicosapentaenoic acid	7α-OHCone	7α-hydroxycholestenone
**EPA-1-G**	EPA-1-glycerol	7-KC	7-ketocholesterol
**EPA-2-G**	EPA-2-glycerol	7-OHC	7-hydroxycholesterol
**EPEA**	*N*-eicosapentaenoylethanolamine		
**LEA**	*N*-linoleylethanolamine		
**OEA**	*N*-oleoylethanolamine		
**PEA**	*N*-palmitoylethanolamine		

## Supplementary Materials

The following are available online at https://www.mdpi.com/2073-4409/9/5/1247/s1. Figure S1. *Napepld* deletion is specific to hepatocytes. (**A**) Localization of *Napepld* mRNA by in situ hybridization in the liver (scale bar: 100 µm; *n* = 4–5). (**B**) eCBome mediator levels in the liver (fmol/mg; *n* = 8–10). (**C**) Specific markers of NPC (*F4/80, Cd31, Ck19* and *Acta2*) and hepatocytes (*Hnf4a*) measured by real-time qPCR (*n* = 4–5). Blue: WT ND mice. Pink: *Napepld*^∆Hep^ ND mice. Data are presented as the mean ± s.e.m. * and *** indicate a significant difference versus WT ND (Respectively *p* < 0.05 and *p* < 0.001) according to the *t*-test. Data with different superscript letters are significantly different (*p* < 0.05) according to a two-way ANOVA followed by a Tukey post hoc test. Lipid abbreviations: 1/2-DPG; 1/2-DPA-glycerol; 1/2-EPG, 1/2-EPA-glycerol; 1-PG, 1-palmitoylglycerol; 2-PG, 2-palmitoylglycerol; DPA-ω3, DPA-omega3. Figure S2. *Napepld*^∆Hep^ mice develop a high-fat diet-like phenotype upon normal diet. (**A**) Plasma insulin levels (pM) at 30 min before and 15 min after oral glucose loading. (**B**) Mean area under the curve (AUC) of insulin measured between 30 min before and 15 min after oral glucose loading. Blue: WT ND mice. Pink: *Napepld*^∆Hep^ ND mice (*n* = 8–10). Data are presented as the mean ± s.e.m. * indicate a significant difference versus WT ND (*p* < 0.05) according to *t*-test. Figure S3. *Napepld*^∆Hep^ mice are more sensitive to liver lipid accumulation. (**A**) Liver triglycerides (nmol/mg). (**B**) Plasma triglycerides (mM). (**C**) Plasma cholesterol (mM). (**D**) Plasma NEFA (mM). Blue: WT ND mice. Pink: *Napepld*^∆Hep^ ND mice (*n* = 8–10). Data are presented as the mean ± s.e.m. Figure S4. Deletion of *Napepld* partially accentuates the obese phenotype induced by a high-fat diet. (**A**) Plasma glucose profile (mg/dl) measured between 30 min before and 120 min after glucose loading. (**B**) Mean area under the curve of glycemia measured between 30 min before and 120 min after glucose loading. (**C**) Plasma insulin levels (pM) at 30 min before and 15 min after glucose loading. (**D**) Mean area under the curve (AUC) of insulin measured between 30 min before and 15 min after glucose loading. (**E**) Insulin resistance index determined by multiplying the AUC of blood glucose by the AUC of insulin. (**F**) Plasma triglyceride concentration (mM). Dark blue: WT HFD mice. Dark pink: *Napepld*^∆Hep^ HFD mice (*n* = 11). Data are presented as the mean ± s.e.m.
