# Supplementary material for: Hepatic NAPE-PLD Is a Key Regulator of Liver Lipid Metabolism
Source: Cells. 2020 May 18;9(5):1247. doi: 10.3390/cells9051247 (PMC7291298; doi:10.3390/cells9051247)
Supplement: Supplementary file 1 [file cells-09-01247-s001.pdf]

***In situ* hybridization**. This technique was performed in collaboration with Creative Bioarray (Shirley, New York, USA). Paraffin-embedded liver tissue sections were pretreated by immersing the slides in diverse xylene, ethanol baths (100%, 85%, 75%) and in pre-warmed 3% H_2O_2. Sections were next pretreated for the protease by being immersed successively in 0.2M HCl, 0.5% Triton X-100 in PBS, 20 µg/mL Proteinase K, 4% PFA baths and finally dehydrated. For the hybridization, *Napepld* probe solution (RNA ISH Probe from Creative Bioarray labelled with digoxin) was added to the hybridization buffer (1/50). Sections were incubated with 200µL of this buffer in a humidified chamber, in the dark at 37 °C for 20 hours. After washing, sections were incubated for 1 hour at 37 °C with Horseradish Peroxidase (HRP)-digoxin conjugate solution. Detection was mediated by diaminobenzidine (DAB). Slides were dehydrated and covered. Bright field images were acquired using a Nikon 80I microscope equipped with a 40× objective.

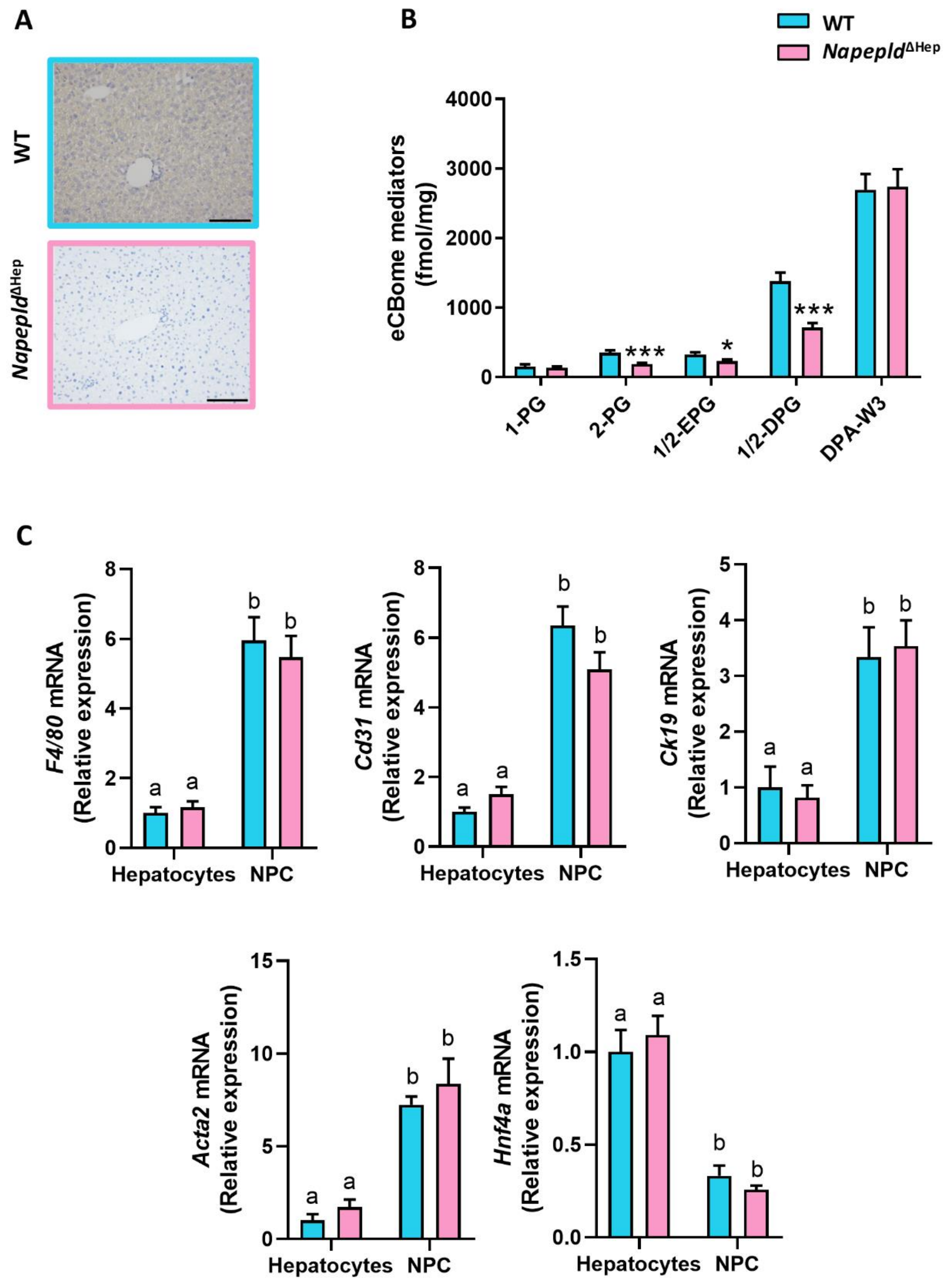

Figure S1. *Napepld* deletion is specific to hepatocytes. (**A**) Localization of *Napepld* mRNA by *in situ* hybridization in the liver (scale bar: 100 µm; n = 4–5). (**B**) eCBome mediator levels in the liver (fmol/mg) (n = 8–10). (**C**) Specific markers of NPC (*F4/80, Cd31, Ck19* and *Acta2*) and hepatocytes (*Hnf4a*) measured by real-time qPCR (n = 4–5). Blue: WT ND mice. Pink: *Napepld*$^{\Delta Hep}$ ND mice. Data are presented as the mean ± s.e.m. * and *** indicate a significant difference versus WT ND (Respectively $p < 0.05$ and $p < 0.001$) according to t-test. Data with different superscript letters are significantly different ($p < 0.05$) according to two-way ANOVA followed by Tukey post hoc test. Lipid abbreviations: 1/2-DPG; 1/2-DPA-glycerol; 1/2-EPG, 1/2-EPA-glycerol; 1-PG, 1-palmitoylglycerol; 2-

PG, 2-palmitoylglycerol; DPA-ω3, DPA-omega3.

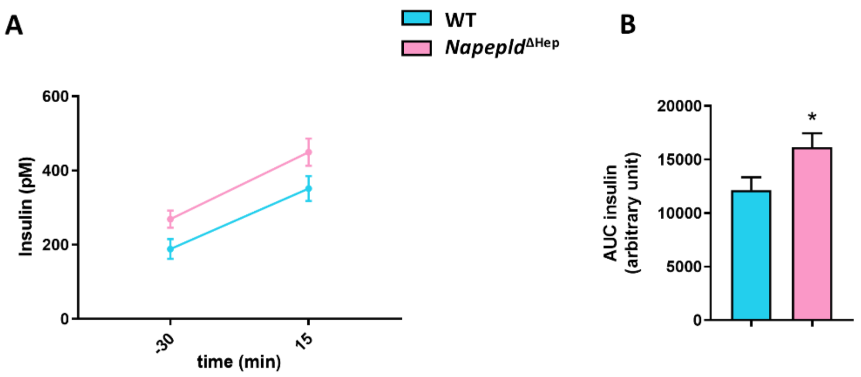

Figure S2. *Napepld*$^{\Delta Hep}$ mice develop a high-fat diet-like phenotype upon normal diet. (**A**) Plasma insulin levels (pM) at 30 min before and 15 min after oral glucose loading. (**B**) Mean area under the curve (AUC) of insulin measured between 30 min before and 15 min after oral glucose loading. Blue: WT ND mice. Pink: *Napepld*$^{\Delta Hep}$ ND mice (n = 8–10). Data are presented as the mean ± s.e.m. * indicate a significant difference versus WT ND ($p < 0.05$) according to t-test.

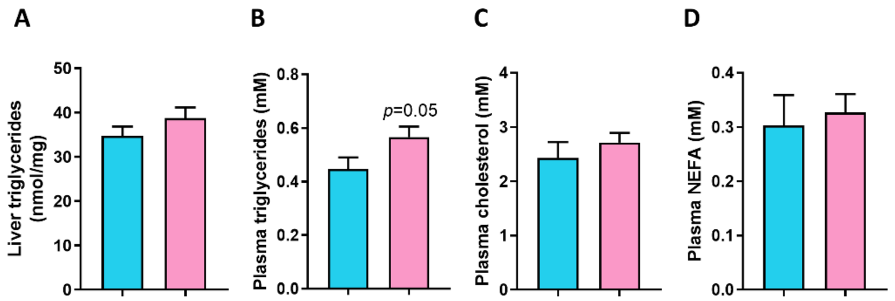

Figure S3. *Napepld*$^{\Delta Hep}$ mice are more sensitive to liver lipid accumulation. (**A**) Liver triglycerides (nmol/mg). (**B**) Plasma triglycerides (mM). (**C**) Plasma cholesterol (mM). (**D**) Plasma NEFA (mM). Blue: WT ND mice. Pink: *Napepld*$^{\Delta Hep}$ ND mice (n = 8–10). Data are presented as the mean ± s.e.m.

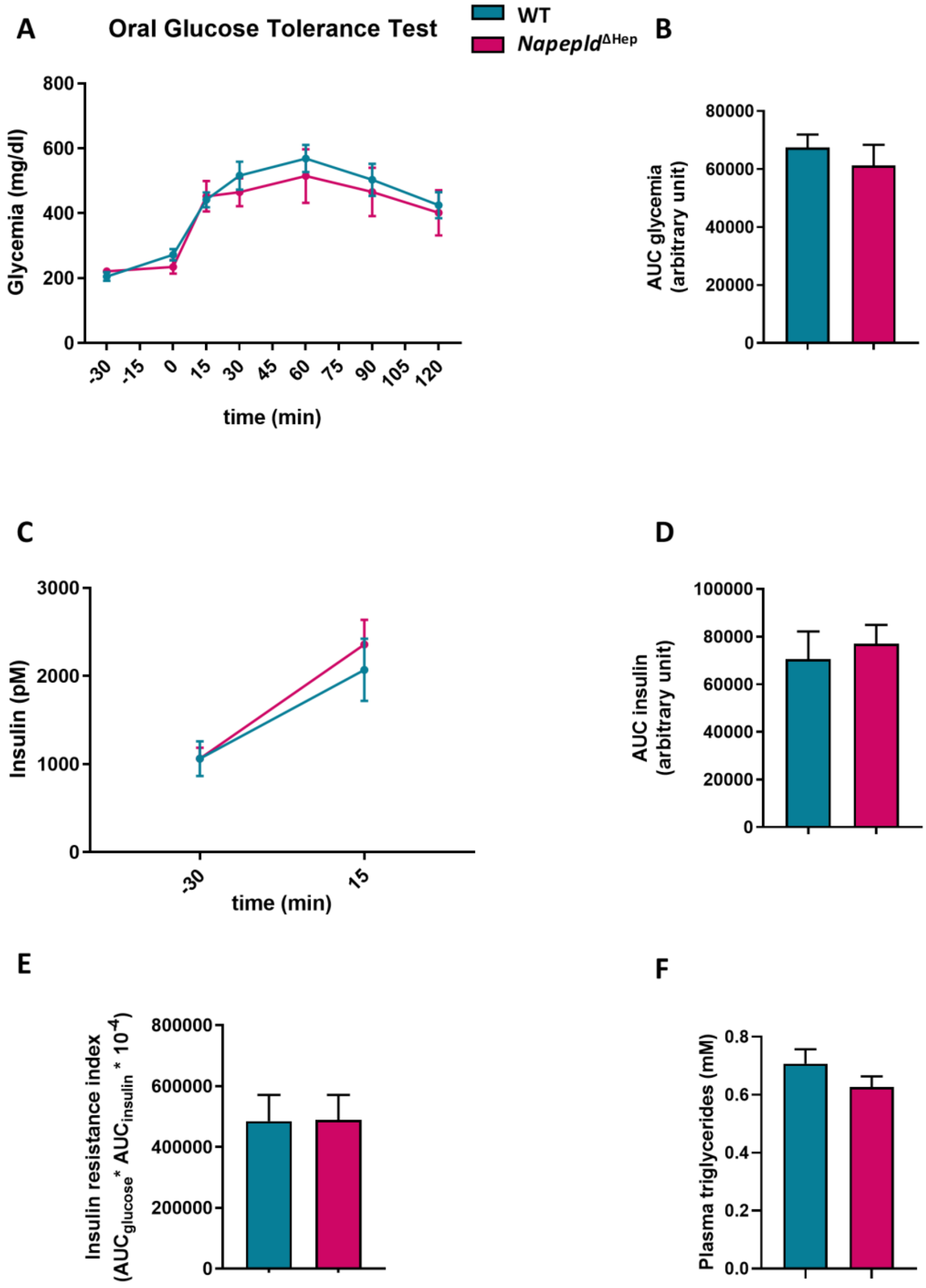

Figure S4. Deletion of *Napepld* partially accentuates the obese phenotype induced by a high-fat diet. (**A**) Plasma glucose profile (mg/dl) measured between 30 min before and 120 min after glucose loading. (**B**) Mean area under the curve of glycemia measured between 30 min before and 120 min after glucose loading. (**C**) Plasma insulin levels (pM) at 30 min before and 15 min after glucose loading. (**D**) Mean area under the curve (AUC) of insulin measured between 30 min before and 15 min after glucose loading. (**E**) Insulin resistance index determined by multiplying the AUC of blood glucose by the AUC of insulin. (**F**) Plasma triglyceride concentration (mM). Dark blue: WT HFD mice. Dark pink: *Napepld*$^{\Delta Hep}$ HFD mice (n = 11). Data are presented as the mean ± s.e.m.
